# Potential of Aqueous Ozone to Control Aflatoxigenic Fungi in Brazil Nuts

**DOI:** 10.5402/2013/859830

**Published:** 2013-07-17

**Authors:** Otniel Freitas-Silva, Héctor Morales-Valle, Armando Venâncio

**Affiliations:** ^1^Institute for Biotechnology and Bioengineering IBB, Centre of Biological Engineering, Universidade do Minho, Campus de Gualtar, 4710-057 Braga, Portugal; ^2^EMBRAPA Food Technology, Avenida das Américas 29501, 23020-470 Rio de Janeiro, RJ, Brazil

## Abstract

This study aimed to verify the use of aqueous ozone as alternative technology for fungal control. Brazil nuts sterilized were inoculated with either 1 × 10^6^ or 1 × 10^7^ conidia mL^−1^ of *Aspergillus flavus* (MUM 9201) to determine optimal treatment parameters and different aqueous ozone contact times. These assays showed that the effect of ozone is almost immediate against *A. flavus*, and the optimum ozone concentration depended on the number of initial viable spores on the shell. The remaining viable spores in the ozone solution were recorded, and the rate of inactivation for each treatment was determined by assessing the ratio between the cfu of each treatment and the control. The ozonized nuts were also cultured to recover the fungal population. Aqueous ozone was effective in reducing the conidia of *A. flavus* and the natural fungal population associated with Brazil nuts. Aqueous ozone presented a great potential to reduce microorganisms counts in Brazil nuts with a great potential use in packing houses for decontamination step.

## 1. Introduction

The Brazil nut (*Bertholietia excelsa*) is an important nontimber forest product (NTFP) from the Amazon forest. Brazil produces approximately 24% of the total world supply of this nut. Brazil nuts exploitation is an important social and economic activity for people living in forest areas. Besides this, it is an important factor in forestry conservation and environmental sustainability [1].

Despite the positive nutrition and health-related aspects of Brazil nuts, they are susceptible to colonization by mycotoxin-producing fungi and consequently to contamination with mycotoxins. There are reports of the presence of *Aspergillus flavus*, and *A. nomius,* which are the main producers of aflatoxins (AF) [2], and more recently two other aflatoxigenic species *A. bombycis and A. arachidicola* [3] and a new nonaflatoxigenic species *A. bertholletius *[4], all of them from this same section, were isolated and identified in Brazil nut samples. 

EU countries and the United States have been the major importers of in-shell and shelled Brazil nuts, respectively. AF contamination constitutes not only an economic problem for Brazil nut producing countries but also a serious health risk for consumers all over the world [5].

Both industries and producers have been making considerable efforts in the past 15 years to minimize fungal growth and AF contamination of tree nuts. Particularly in the case of Brazil nuts, the climate conditions in the Amazon environment and the characteristics of the exploitation activity (collecting and primary handling) cannot be controlled, exerting direct or indirect effects on the toxigenic fungi and on AF or multitoxin production [6].

Processes and/or treatments that have been proven to reduce AF levels in Brazil nuts include shelling and sorting by size, specific gravity, color, or damage [1]. Furthermore, several strategies have been reported for the management of fungi and mycotoxins already present in raw material and food [7]. Of the available methods to ensure food safety, ozone application is one of the most promising ones. Ozonation is a chemical means of food processing that involves exposing food or an intermediate product to O_3_ [7, 8]. Ozone can be applied as a gas or dissolved in an aqueous solution. The first method is more useful, but both have been used successfully on many food products to reduce (a) postharvest diseases [9], (b) viability of potential mycotoxin-producing fungi, and (c) mycotoxin accumulation [7].

The fact that ozonation is a nonresidual treatment, an advantage in light of the increasing concern related to the use of chemicals on food chains, makes it a promising method to treat Brazil nuts. The assays performed in the present work are focused on the use of aqueous ozone since it is known to be a strong antimicrobial agent [7, 10] and its use for mycotoxin destruction has been studied earlier [11]. Also, ozone is generally recognized as a safe (GRAS) product, and it has been already used in many agricultural products, including organically labeled ones [12], like Brazil nuts. In view of this, the present work aimed to investigate the potential of aqueous ozone to control the fungi associated with Brazil nuts, focusing on *Aspergillus flavus*.

## 2. Materials and Methods

The samples used were collected from the 2009 Brazil nuts harvest, obtained from an agroforestry production area in Pará state, Brazil. The initial sample weighed about 40 kg and was homogenized to obtain a working sample.

The working samples of Brazil nuts were divided into two groups: (i) naturally contaminated nuts (without sterilization) and (ii) sterilized nuts intended for studies with artificially contaminated nuts. For the latter group, the sterilization was carried out by autoclaving at 121°C during 20 min at 1 atm, and the contamination was with the MUM 9201 strain of *A. flavus*. This strain is a well-known producer of both AF and cyclopiazonic acid (CPA) [13, 14].

To obtain the stock solution, water saturated with ozone was prepared by bubbling gas, generated by the passage of extra dry oxygen through a reactor (Anseros, Model CD-COM-HF-4) for 15 minutes (power generator at 100%; gas flow of 25 Lh^−1^) in a bottle with 1000 mL of water, at 3°C. The final aqueous ozone concentration was determined by the colorimetric method with a JASCO V560 spectrophotometer (*λ*_max_ = 258 nm and *ε* = 2900 M^−1^ cm^−1^) [15]. The ozone concentration in the stock solution ranged from 25 to 40 mg/L.

The different ozone concentrations required for the assays were obtained from the stock solution diluted with ozone demand-free water as necessary, until obtaining the ozone working dilution. Nonozonated water was used for control treatments. Only purified water obtained by successive reverse osmosis by Milli-Q plus system (Millipore, Molsheim, France) was used in the tests. 

A MUM 9201 suspension of 1 × 10^7^ conidia × mL^−1^ was prepared in peptone water (0.1%). From that, a 10x diluted conidial suspension was prepared and the number of viable conidia of both suspensions was assessed by inoculation on malt extract agar (MEA: malt extract, 20 g/L; glucose, 20 g/L; peptone, 1 g/L, agar, 20 g/L).

The high-concentrate conidial solution (HC) yielded about 400 viable conidia, whereas the low-concentrate suspension (LC) yielded about 40. From now on all the assays are based on the number of viable conidia.

### 2.1. Effect of Ozonation Time on Viable Conidia

Suspensions of *A. flavus (*MUM 9201) with 0.1 mL of either HC or LC conidia were washed in 20 mL of ozone solution for 2.5 min, 5 min, 10 min, 20 min, 30 min, or 60 min. Subsequently, the ozone solution was filtered in a Millipore filtration system. Filters (0.2 *μ*m) with washed conidia were plated directly on MEA and incubated at 25°C for 3 days for assessment of colony-forming units (cfu). Control tests were carried out by filtration of each conidial suspension in a solution of Tween 80 in Milli-Q water (0.005% v/v). The duration of the ozone treatment was controlled by the addition of 1 mL of 1 M reagent-grade sodium formate solution to neutralize the remaining dissolved ozone after the desired contact time [16]. Three replications of each treatment were carried out, and the results underwent full factorial testing at *α* < 0.05. In order to assess whether the factor time was affected by the ozone concentration (interaction between effects), the whole assay was carried out at four different ozone concentrations (0, 1, 10, and 20 mg L^−1^). In summary, the assay consisted of two different conidial concentrations (HC or LC), six submersion times (2.5, 5, 10, 20, 30, and 60 min), four ozone concentrations (0, 1, 10, and 20 mg L^−1^), and three replications (2 × 6 × 4 × 3 = 144 experiments).

### 2.2. Effect of Ozone Concentration on Recovery of Viable Conidia from Brazil Nuts

Six sterilized nuts were inoculated with 0.1 mL of either the LC or HC conidial solution of *A. flavus *MUM 9201. The nuts were stored overnight at room temperature. Then the nuts were submerged in 20 mL of ozone solution at different concentrations (1, 5, 10, 15, 20, 25, and 30 mg L^−1^). Control tests were carried out by submerging nuts in a solution of Tween 80 in Milli-Q water (0.005% v/v). After 15 min, 20 mL of the ozone or Tween 80 solution was filtered (0.2 *μ*m filter) in a Millipore filtration system, and the filtrate was plated directly on MEA and incubated at 25°C for 3 days for determination of cfu. The whole experiment was carried out twice, with three replications per assay. The effect of both the ozone concentration and initial conidial concentration on the viable spores after ozonation was studied by full factorial tests at *α* < 0.05.

### 2.3. Effect of Ozonation on Shell Color

The effect of the ozonation on the color of the nutshell was tested. For that purpose, 20 nuts were submerged in aqueous ozone during 30 min. Five different ozone concentrations were tested (0, 0.1, 1.0, 10, and 20 mg L^−1^). The control sample consisted on 20 untreated nuts chosen randomly. Chromaticity values of the shells in the *L* ^*^ *a* ^*^ *b* ^*^ space coordinates were recorded [17]. The values of *L* ^*^ (lightness or brightness) varied from black (0) to white (100), and the chroma values of *a* ^*^ ranged from green (−60) to red (+60) and *b* ^*^ ranged from blue (−60) to yellow (+60). The hue [defined as arctan(*b* ^*^/*a* ^*^)] was calculated. An Avantes spectrometer (AvaMouse 2.0, Eerbeek, Netherland) was used. The change in shell color was evaluated by comparing the parameters hue and *L* of the control samples with those of the treated ones. The significance of the results was assessed by *t*-tests for independent samples at *α* < 0.05.

### 2.4. Assays with Inoculated and Natural Contaminated Nuts

In order to apply the correct ozone concentration during the treatment, it was necessary to know the degree of contamination of the nutshell. For that purpose, a previous assay with artificially contaminated nuts was carried out to learn the percentage of viable conidia that migrate from the nutshell to an aqueous solution. For this purpose, 100 *μ*L of either the LC or HC conidial suspension was inoculated onto the sterilized nut surface. After one hour at room temperature, the nut was submerged in 20 mL of a solution of Tween 80 (0.005% v/v) in water. After 1 hour of shaking, this solution was filtered (0.2 *μ*m filter) in a Millipore filtration system, and the filtrate was plated directly on MEA and incubated at 25°C for 3 days for cfu determination. The assay was carried out three times with three replications (three nuts) each time. 

In parallel, to know the degree of contamination of the naturally contaminated nuts, they were submerged in 20 mL of a solution of Tween 80 (0.005% v/v) in water. Just as in the previous assay, after 1 hour shaking, 20 mL of the aqueous solution was filtered (0.2 *μ*m filter), and the filtrate was plated directly on MEA and incubated at 25°C for 3 days for cfu determination. Three tests were performed with three nuts each. The percentage of migration obtained in the previous assay is used to assess the total initial viable conidia on the nutshell.

Next, three more sterilized nuts were inoculated with 100 *μ*L of either the LC or HC conidial solution and left 1 hour at room temperature. In parallel, three naturally contaminated nuts were randomly picked. Each sample was submerged in 20 mL of ozone solution (20 mg L^−1^). Each ozone solution was filtered (0.2 *μ*m filter) in a Millipore filtration system and the filtrate was plated directly on MEA. The determination of cfu was carried out after 3 days at 25°C. The whole assay was carried out three times. The fungi isolated from the naturally contaminated samples were identified by genus according to the identification key of Pitt and Hocking [18].

The significance of the efficiency of ozonation was assessed by comparison of viable conidia in aqueous solution to viable conidia in ozone solution by means of *t*-test for separate samples at *α* < 0.05.

## 3. Results

Neither the duration of aqueous ozone treatment nor the interaction with the ozone concentration affected the efficiency of the treatment.  Table 1 shows the percentage of viable spores after different treatment durations. However, the ozone concentration factor as a single effect was highly significant (*P* < 0.001) and so was further tested (see below). Therefore, the effect of the ozone against the spores was instantaneous at all ozone concentrations. This was corroborated by comparing the results of an assay in which sodium formate solution was used with the results of another assay in which the treatment duration was determined only by the time of submersion of the nuts in the ozone solution (data not shown). Thereafter, the sodium formate solution was no longer used, and the duration of the treatment (which influences the shell color) was studied 

**Figure 1:**
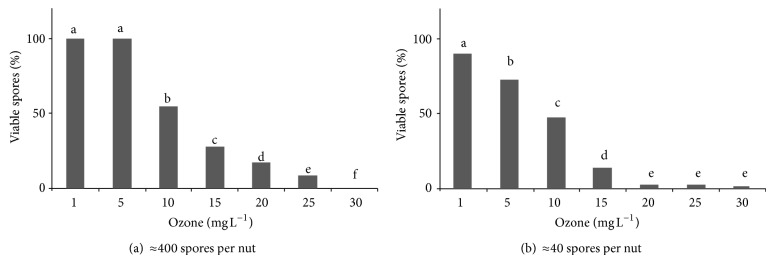
Percentiles of *A. flavus* viable conidia recovered after aqueous ozone exposure up to 30 mg L^−1^ in high (a) and low (b) concentration of conidia per nut.

The effect of the ozone concentration on the treatment efficiency was highly significant (*P* < 0.001). In general, whatever the initial charge of spores, the higher the ozone concentration, the lower the final concentration of viable conidia (Figure 1). 

However, the interaction of ozone concentration with initial viable spores was also highly significant (*P* < 0.001). This interaction showed that for lower initial viable spore charge it was enough to use an ozone concentration of 20 mg L^−1^, as no significantly better results were observed with higher concentrations.

The submersion of LC or HC inoculated nuts in a solution of Tween 80 showed that, whatever the initial number of viable spores (HC or LC), about 60% of them migrated to the water solution. The submersion of naturally contaminated nuts in the Tween 80 solution yielded 32 ± 5 conidia per nut. This means the initial contamination was 53 ± 9 conidia per nut. According to the results obtained with different ozone concentrations (Figure 1), a concentration of 20 mg L^−1^ of ozone was chosen to assay with HC and LC inoculated nuts and naturally contaminated nuts. The most frequently isolated filamentous fungi from the naturally contaminated nuts were, in descending order, *Aspergillus* section *Nigri*, *Cunninghamella* sp, *Penicillium* spp., and *Aspergillus *section *Flavi*, with incidences of 26%, 21%, 16%, and 5%, respectively. The rest of the fungal isolates (33%) were yeasts.

**Figure 2:**
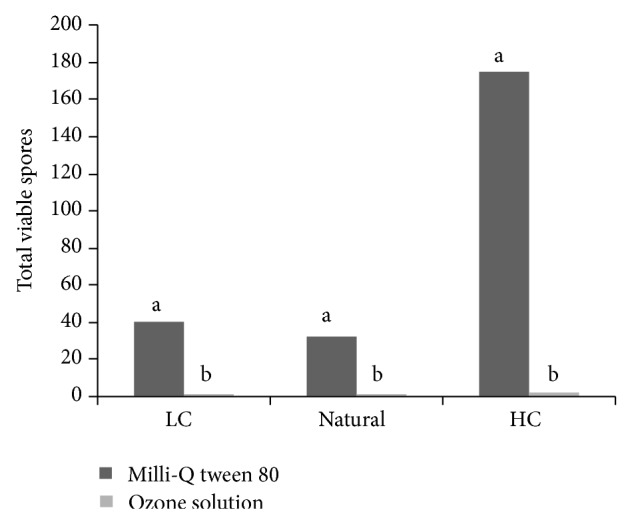
Reduction of viable spores on Brazil nuts from natural population and inoculated *A. flavus* (MUM 9201) after aqueous ozone 20 mg mL^−1^. The low-concentrate suspension (LC) and the high-concentrate conidial solution (HC).

The results of submersion of (a) nuts artificially inoculated with LC solution of *A. flavus *MUM 9201, (b) naturally contaminated nuts, and (c) nuts artificially contaminated with HC suspension of *A. flavus *MUM 9201 in a 20 mg L^−1^ ozone solution showed almost 0 viable spores after treatment in cases (a) and (b) and about 3 viable spores per nut in case (c) (Figure 2). In all cases, the reduction of viable spores was highly significant (*P* < 0.001).

**Figure 3:**
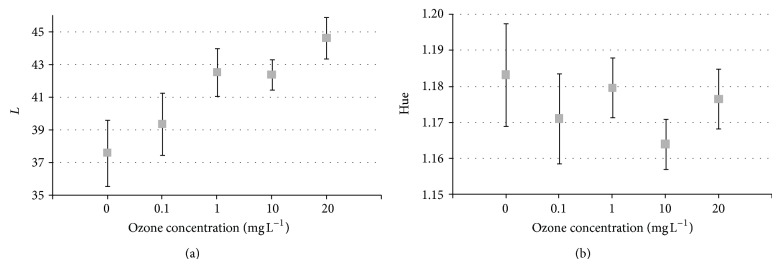
Effect of aqueous ozone concentration on color parameters (a) *L* and (b) hue.

The influence of the aqueous ozone on the shell color was evident at higher ozone concentrations. The values of the parameter *L* increased with rising ozone concentration and significantly differed from the control ones at concentrations higher than 10 mg L^−1^. On the other hand, the hue values decreased with increasing ozone concentration and significantly differed from control ones at concentrations of 10 mg L^−1^ and higher (Figure 3).

## 4. Discussion

The genus* Aspergillus* is mainly responsible for fungal contamination and subsequent production of AF in the field, at harvest, and during postharvest operations and storage of nuts [13]. Due to high potential risk of nut contamination, decontamination methods are of great interest from economic, public health, and environmental aspects. Improving postharvest processing followed by further prevention of fungal growth with ozone treatment is one of the most effective ways to restrict AF contamination and would go a long way to reduce health-related risks from eating contaminated nuts and economic losses during production [19].

This work focused on fungal decontamination of Brazil nuts using different ozone concentrations. The use of ozone in dried fruits and nuts has been studied before. The contamination of dried figs using 0.01 to 0.02 g m^3^ of gaseous O_3_ for 3 and 4 h resulted in the reduction of total mycobiota counts [20]. When the efficiency of gaseous ozone was evaluated for the degradation of AF on pistachio kernels and ground pistachios [21], pistachios were contaminated with known concentrations of AFB_1_, AFB_2_, AFG_1_, and AFG_2_, and then samples were exposed to gaseous ozone at 5.0, 7.0, and 9.0 g m^−3^ concentrations for 140 and 420 min at 20°C and 70% RH. This works although presented a significant reduction in total AF and in AFB_1_ for pistachio kernels, no significant change in the fatty acid composition was observed. Likewise, no significant changes were found in sweetness, rancidity, flavor, appearance and overall palatability between ozonated and nonozonated pistachio kernels.

In Brazil nuts, other authors have studied the potential of gaseous ozone. In research conducted by Giordano and coauthors [22] they had shown that O_3_ treatment for 5 h at 31 mg L^−1^ inhibited the viability of fungi of the nut-contaminating mycobiota, including the aflatoxigenic *Aspergillus*. In another work [23] also was reported the potential of gaseous ozone to treat Brazil nuts and considered the best method, especially when associated with vacuum. According to these authors, ozone treatment was able to (a) degrade AF (15 *μ*g kg^−1^), (b) reduce fungi and yeast spores from 1.8 × 10^4^ cfu g^−1^ to no growth, (c) keep the fatty acid oxidation indicator (i.e., malondialdehyde) stable, and (d) even improve the sensory attributes for consumer acceptance. Such features are of great importance for the gaseous ozone treatment to be considered a good sanitizer. Unfortunately, the use of gaseous ozone in some cases could cause toxicity since the concentrations of gaseous O_3_ necessary to inactivate the conidia of *Penicillium digitatum*, *P. italicum*, and *Botrytis cinerea* from table grapes were relatively high. The need for such high O_3_ concentration makes it hard to achieve complete containment of the gas, and thus the worker safety may be jeopardized [24]. Gaseous ozone treatment needs some adjustments in its application to comply with regulations since O_3_ concentrations cannot exceed 0.075 *μ*LL^−1^ during an 8 h workday [25]. 

According to our results, the reaction to the ozone is almost instantaneous. The application of aqueous ozone treatments in industries does not seem to be very complicated. It is necessary to learn the degree of contamination of the nuts in advance, because if it is not very high, lower ozone concentrations can be used. For the same reason it is necessary to learn which degree of contamination is acceptable after treatment, because higher ozone concentrations will be required if lower levels of spores per nut are to be achieved.

Although the effect of gaseous ozone on red pepper treated with concentrations up to 66 g·m^3^ for up to 1 hour did not significantly change the product's color [26], it seems that some changes could happen in the case of Brazil nuts. Such effect could depend on the ozone concentration as well as the submersion time. Taking into account that the effect of ozone against conidia seems to be immediate, it seems feasible to reduce color changes by minimizing as much as possible the submersion time and adjusting the ozone concentration to the minimum needed for the initial degree of contamination. 

When the effectiveness of gaseous and aqueous ozone on mycobiota and AFB_1_ content of dried figs [27] was studied, in both treatments, degradation of AFB_1_ increased with increasing ozonation time. Long gaseous ozone treatment, as mentioned previously, is hard to control and can jeopardize worker safety. These authors [27] stated that gaseous ozone was more effective than ozonated water for reduction of AFB_1_, whereas ozonated water was more effective for decreasing microbial counts.

Our results show that the use of aqueous ozone is a viable alternative technique to control fungi on Brazil nuts, showing optimum control of a large spectrum of fungi and good applicability to complex matrices such as nuts. The aqueous O_3_ solution can also be recommended to control other mycotoxin-producing Aspergilli. However, additional experiments are needed to adjust the method for use in Brazil nut packing houses.

Food-related application of ozone is currently restricted in packing houses, especially for leaf and fruit sterilization [7]. The food processing industry constantly searches for new technologies to improve commercial sterilization of food commodities, especially those associated with mycotoxins and other hazards.

As shown in this paper, aqueous ozone application with accurate O_3_ dosification can be an efficient method for disinfection of dry food surfaces. O_3_ disinfection of food surfaces opens up an alternative for the elimination of fungal contamination since ozone application on food is easy and, according to the results shown here, effective at low doses and short durations, yielding no residues on the product or in the environment, for which reason it has GRAS certification [12].

More research must be undertaken to evaluate this technique to become common in the food industry, to determine the ideal concentration and projected cost of large-scale treatment of Brazil nuts.

## Figures and Tables

**Table 1:** Viable conidia of *A. flavus* (MUM 9201) after different treatment duration and aqueous ozone concentration.

	Time (min)	Viable conidia (%) after different time exposure (min) to ozone					
		2.5 (±sd)	5 (±sd)	10 (±sd)	20 (±sd)	30 (±sd)	60 (±sd)
Ozone (mg L^−1^)	0	96 ± 7	94 ± 2	92 ± 0	93 ± 5	97 ± 5	94 ± 3
	1	51 ± 1	69 ± 2	64 ± 20	59 ± 4	58 ± 7	61 ± 3
	10	7 ± 4	5 ± 2	6 ± 6	5 ± 3	3 ± 2	2 ± 2
	20	2 ± 3	1 ± 0	2 ± 1	2 ± 1	1 ± 1	3 ± 1
